# Data on enhanced expression and purification of camelid single domain antibodies from *Escherichia coli* classical inclusion bodies

**DOI:** 10.1016/j.dib.2017.03.039

**Published:** 2017-03-31

**Authors:** Maristella Maggi, Claudia Scotti

**Affiliations:** Department of Molecular Medicine, Unit of Immunology and General Pathology, University of Pavia, Via Ferrata, 9, 27100 Pavia, Italy

**Keywords:** Single domain antibody, VHH, sdAb, Protein purification, Classical inclusion bodies, Non-classical inclusion bodies, Urea extraction, Arginine extraction

## Abstract

Heterologous expression of high amounts of recombinant proteins is a milestone for research and industrial purposes. Single domain antibodies (sdAbs) are heavy-chain only antibody fragments with applications in the biotechnological, medical and industrial fields. The simple nature and small size of sdAbs allows for efficient expression of the soluble molecule in different hosts. However, in some cases, it results in low functional protein yield. To overcome this limitation, expression of a 6xHistag sdAb was attempted in different conditions in *Escherichia coli* BL21(DE3) cells. Data showed that high amount of sdAb can be expressed in *E. coli* classical inclusion bodies, efficiently extracted by urea in a short-time, and properly purified by metal ion affinity chromatography. These data originate from the research article "Enhanced expression and purification of camelid single domain VHH antibodies from classical inclusion bodies" Maggi and Scotti (2017) [1] (DOI: http://dx.doi.org/10.1016/j.pep.2017.02.007).

**Specifications Table**

**Table:** 

Subject area	*Molecular Biology*
More specific subject area	*Protein expression and purification*
Type of data	*Images of SDS-PAGE gels or blot, and histograms.*
How data was acquired	*FPLC, SDS-PAGE electrophoresis, western blot and bicinchoninic acid assay*
Data format	*Analyzed*
Experimental factors	*sdAb gene cloning and transformation in expression host. Protein over-expression by autoinducing or by IPTG. Inclusion bodies isolation. Protein extraction from cytosolic or insoluble fraction. Affinity, ion exchange and size exclusion chromatography.*
Experimental features	*Optimization of protein expression and purification*
Data source location	*University of Pavia, Pavia, Italy*
Data accessibility	*Data is with this article.*
Related research article	*Enhanced expression and purification of camelid single domain VHH antibodies from classical inclusion bodies*. DOI: http://dx.doi.org/10.1016/j.pep.2017.02.007

**Value of the data**

• The data indicates that camelid sdAb can be expressed in *Escherichia coli* both in cytosol or in inclusion bodies at 37 °C.
• The data shows that expression and purification is more efficient when the protein is expressed in the form of classical inclusion bodies.
• The data obtained from optimization of urea-mediated protein extraction from classical inclusion bodies indicates that complete protein extraction can be obtained in 4 h.

## 1 Data

**Fig. 1:**
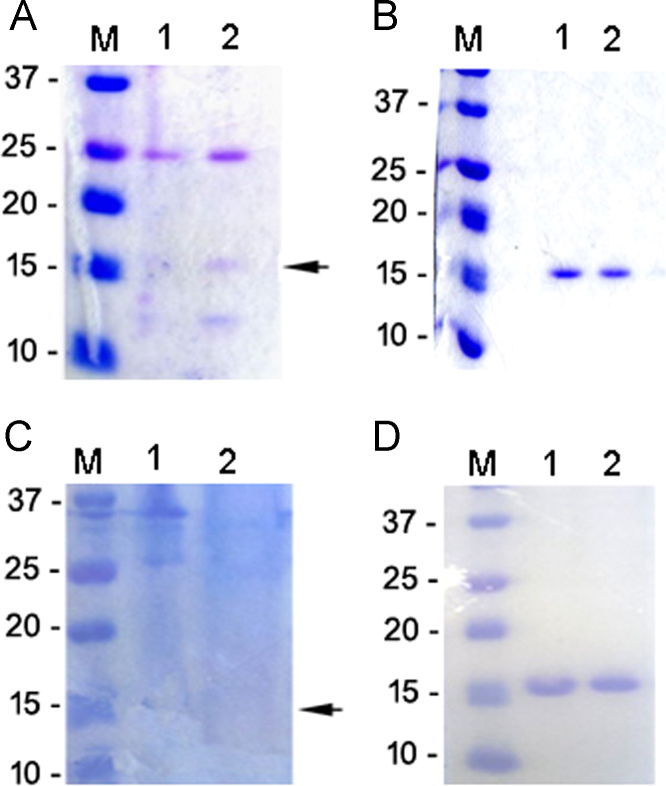
SDS-PAGE analysis of sdAb expressed in different conditions. Panel A: partially purified sdAb expressed by auto-induction and purified by IMAC. Lane M: MW standards (BioRad precision Plus), lanes 1 and 2: IMAC fractions. Panel B: fully purified sdAb expressed by IPTG and purified by IMAC and anion exchange. Lane M: MW standards (BioRad precision Plus), lanes 1 and 2: unbound fraction after anion exchange at pH 7.4. Panel C: protein extracted from non classical inclusion bodies by arginine. Lane M: MW standards (BioRad precision Plus), lane 1 soluble fraction, lane 2: non classical inclusion bodies extracted fraction. Panel D: fully purified sdAb expressed in classical inclusion bodies and extracted by urea. Lane M: MW standards (BioRad precision Plus), lane 1 e 2: refolded fraction after IMAC.

**Fig. 2:**
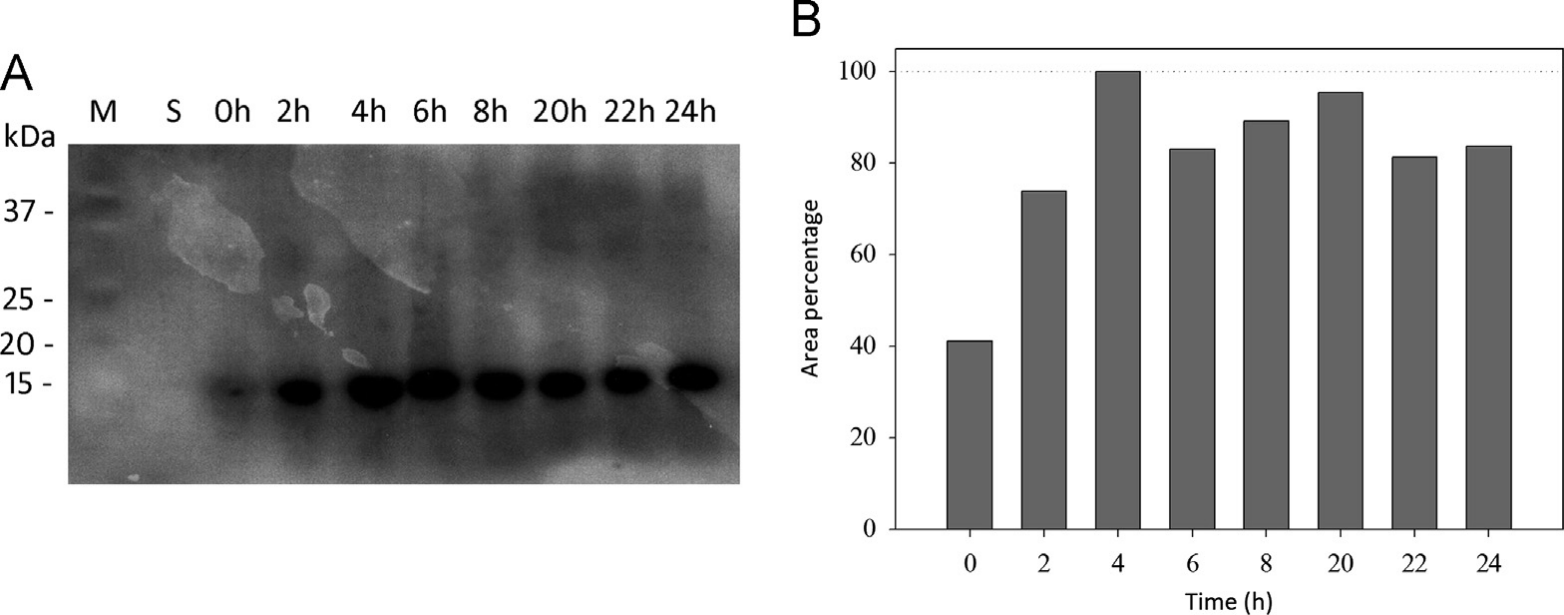
Classical inclusion bodies (cIBs): sdAb extraction kinetic. Panel A: Western-blot results of the time course of urea-extracted sdAb. M: Precision Plus protein standard (BioRad); S: soluble fraction; 0–24 h: time points. Panel B: ImageJ quantitative analysis of Panel A indicating sdAb expression at each time-point. Peak areas are reported in percentage versus the highest value (lane n. 4) and indicate the relative amount of each band positive to the western blot.

**Fig. 3:**
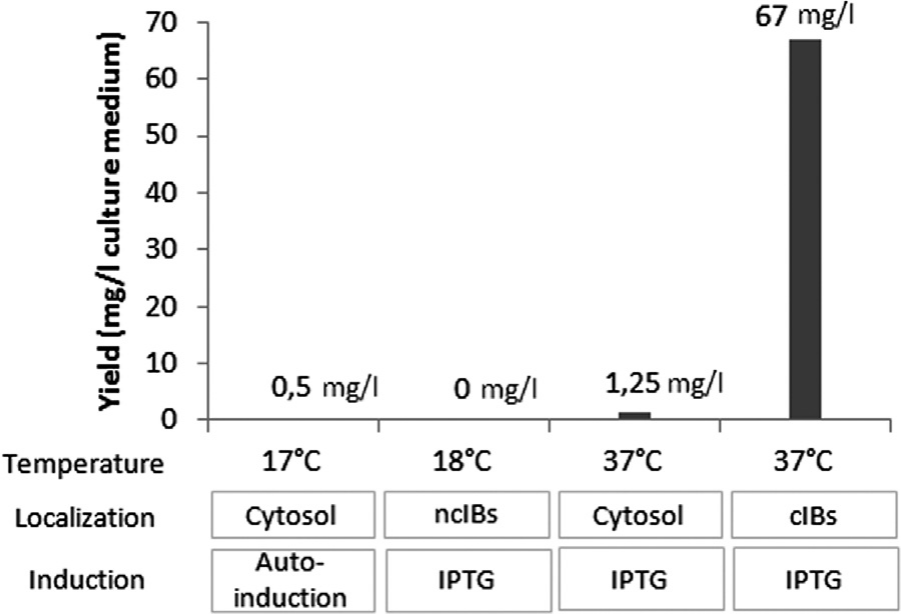
sdAb purification yields.

The dataset of this article provides comparison of expression and purification yields of 4 different methods used to produce a recombinant single domain antibody (sdAb) [1]. In particular, the target protein expression was attempted at different temperatures (e.g., 17 °C, 18 °C or 37 °C), in the cytosolic and insoluble fraction and using two induction methods (e.g., autoinducing and isopropil-β-D-1-thiogalactopyranoside, IPTG). Fig. 1 shows SDS-PAGE of sdAb expression comparing: the cytosolic expression by autoinducing (panel A), the cytosolic expression by IPTG induction (panel B), the expression in non classical inclusion bodies (ncIBs, panel C), and the expression in classical inclusion bodies (cIBs, panel D). Fig. 2 shows sdAb extraction kinetics from classical inclusion bodies and Fig. 3 recapitulates sdAb purification yields.

## 2 Experimental design, materials and methods

### 2.1 sdAb cloning

The sdAb, an engineered version of a previously described [2], was obtained by gene synthesis (GeneArt™). The resulting construct was subcloned into the pET45b(+) expression vector (Novagen) using *Nco* I and *Xba* I as cloning sites for the gene 5′- and 3′-end, respectively. A 6xHis purification tag was inserted at the gene 3′-end. After sequencing of the insert, the vector was transformed into *E. coli* BL21(DE3) *ΔansA/ΔansB* strain kindly provided by Douglas Scott Merrell (University of the Health and Sciences, Bethesda, MD, US) for protein expression. The strain is custom engineered to lack endogenous L-asparaginase I and II encoded by *ansA* and *ΔansB* genes, respectively. Bacterial L-asparaginases (EC 3.5.1.1) are amidohydrolases involved in ammonia metabolism.

### 2.2 Cytosolic protein expression by autoinducing

A starting culture was obtained by inoculating one single clone of *E. coli BL21(DE3) ΔansA/ΔansB* transformed with the expression construct into 50 ml Luria-Bertani (LB) medium containing 100 µg/ml ampicillin (Amp) and 100 µg/ml streptomycin (Str). The former antibiotic is needed to select clones containing the recombinant vector, the latter instead is needed to select the *E. coli* BL21(DE3) *ΔansA/ΔansB* strain. According to Studier [3], 10 ml of overnight pre-inoculum were diluted into 500 ml ZYP-5052 reach medium (10 g/l Tryptone, 5 g/l yeast extract, 50 mM Na_2_HPO_4_, 50 mM KH_2_PO_4_, 25 mM (NH_4_)_2_SO_4_, 1 mM MgSO_4_, 0.5% w/v glycerol, 0.05% w/v glucose, 0.2% w/v α-lactose, pH 6.75) and incubated at 37 °C for 3 h and then at 17 °C for 21 h with vigorous shaking (250 rpm). The next day, cells were collected by centrifugation at 8,000 rpm and at 4 °C, resuspended in buffer A (50 mM Na-phosphate, 300 mM NaCl, 10 mM imidazole, pH 8.0) and processed as described below.

### 2.3 Cytosolic protein expression by IPTG induction

A starting culture was obtained as described above. Eight ml of *E. coli BL21(DE3) ΔansA/ΔansB* containing the vector of interest and grown overnight were diluted in 500 ml LB medium containing 100 µg/ml Amp and 100 µg/ml Str and the culture was incubated at 37 °C with shaking at 250 rpm. At an OD_600_ of 0.6, the culture was induced with 1 mM IPTG (Sigma) for 5 h. After induction, cells were collected by centrifugation at 8,000 rpm and at 4 °C, resuspended in buffer A (50 mM Na-phosphate, 300 mM NaCl, 10 mM imidazole, pH 8.0) and processed as described below.

### 2.4 sdAb purification from cytosolic fraction

The cell suspension obtained after auto- or IPTG-induction was kept on ice throughout all the passages and sonicated using an Omni Sonic Ruptor 400 sonicator (OMNI International) for 4 cycles, each consisting of 60% power for 1 min followed by a 1 min interval. Cell debris were removed by centrifugations, a first one at 14,000 rpm and a second one at 18,000 rpm, and the clear cell extract was 0.22 µm filtered. The cell extract was maintained on ice and loaded at 0.5 ml/min onto a 1 ml HisTrap column (GE Healthcare) equilibrated in buffer A. Bound proteins elution was obtained by an imidazole step gradient (25, 50, 100, 250 and 500 mM imidazole). According to the results of SDS-PAGE, fractions positive for the sdAb were pooled and their buffer was changed to 50 mM Na-phosphate, pH 7.4 before loading them onto a 1 ml HiTrap Q XL column (GE Healthcare). Buffer exchange was obtained using a 26/10 Desalting column (GE Healthcare). Elution of bound proteins after anionic exchange was obtained with 50 mM Na-phosphate, pH 7.4, 1 M NaCl. Fractions positive for the sdAb both on SDS-PAGE and on western blot were analyzed by analytic gel-filtration using a Superdex 75 GL 10/300 column (GE Healthcare) equilibrated in Na-phosphate 50 mM, NaCl 100 mM, pH 7.4. The overall purification yield of the homogenous sdAb solution was determined by assessing protein concentration by the bicinchoninic acid assay method (Pierce™).

### 2.5 sdAb expression in non-classical inclusion bodies (ncIBs)

*E. coli BL21(DE3) ΔansA/ΔansB* cells transformed with the expression vector containing the sdAb insert were grown at 37 °C shaking at 250 rpm overnight. Ten ml of starting culture were diluted in 500 ml LB media containing 100 µg/ml Amp and 100 µg/ml Str. The culture was incubated at 37 °C shaking at 250 rpm and protein expression was obtained by adding 0.4 mM IPTG when OD_600_ reached 0.6. In order to induce non-classical inclusion bodies (ncIBs) formation, the cells were grown at 18 °C with shaking at 250 rpm for 24 h [4]. Cells from 1 l culture were collected by centrifugation and resuspended in 50 ml resuspension buffer A (RA, 50 mM Tris–HCl, 500 mM NaCl, pH 8.5). The cell suspension was subjected to 4 freeze-thaw cycles at −80 °C. After the last cycle, cells were disrupted by sonication (10 cycles of 1 min at 40% power with 1 min interval). The cell lysate was centrifuged and washed three times with ice-cold buffer RA. Washed inclusion bodies were resuspended in 30 ml ice-cold buffer RA added with 2 M L-arginine and a redox couple (5 mM GSH, 0.5 mM GSSG), and incubated in agitation at 4 °C for 72 h. The solution was then diluted 100-times, centrifuged and 0.45 µm filtered. The cleared inclusion bodies extract was loaded onto a 5 ml HisTrap column (GE Healthcare).

### 2.6 sdAb expression in classical inclusion bodies (cIBs)

A starting culture was prepared as reported above and diluted 50 times in LB media containing 100 µg/ml Amp and 100 µg/ml Str. At an OD_600_ of 0.6, 1 mM IPTG was added to induce protein expression. After induction, cells were incubated at 37 °C with shaking at 250 rpm for 5 h. The protocol used for classical inclusion bodies (cIBs) protein extraction was modified from Mahlawat et al. [5]. Cells from 1 l culture were collected by centrifugation and resuspended in 50 ml lysis buffer A (LA, 100 mM Tris–HCl, 10 mM EDTA, pH 8.0). Cell lysis was obtained by 8 cycles of sonication at 40% power each consisting of 1 min sonication and 1 min interval. Inclusion bodies were recovered by centrifugation and washed once with 20 ml washing buffer A (WA, 100 mM Tris–HCl, 10 mM EDTA, 1 M NaCl, pH 8.0), and twice with 20 ml washing buffer B (WB, 100 mM Tris–HCl, 10 mM EDTA, 1% v/v Triton X100, pH 8.0). Washed inclusion bodies were resuspended in 50 ml denaturing buffer (DB, 100 mM NaH_2_PO_4_, 10 mM Tris–HCl, 8 M urea, pH 8.0) and stirred at room temperature for 24 h maximum. In order to evaluate the extraction efficiency, 1 ml samples were withdrawn at different time points. A 100 µl sample of denatured sdAb extracted by urea from classical inclusion bodies (cIBs) was analyzed by size exclusion chromatography (SEC) using a Superdex 75 GL 10/300 column equilibrated in denaturing buffer.

Full purification was done both after 4 and 24 h urea-extraction. The denatured fraction was centrifuged at 25 °C and 0.45 µm filtered; cleared inclusion bodies extract was loaded onto a 1 ml HisTrap column (GE Healthcare) equilibrated in denaturing buffer. Bound proteins were eluted with the same buffer added with 300 mM imidazole. Fractions corresponding to the obtained peak were collected, pooled and roller-incubated in the presence of 60 mM GSH for 1 h. Subsequently, the sample was diluted 80 times in refolding buffer (RB, 50 mM Tris–HCl, 5% v/v glycerol, pH 8.0) added with 0.5 mM GSSG and incubated at 4 °C stirring for 24 h. The refolded fraction was centrifuged, 0.22 µm filtered and loaded onto a 5 ml HisTrap column (GE Healthcare) equilibrated in refolding buffer. Protein elution was obtained by RB supplemented with 500 mM imidazole. The obtained fractions were checked on SDS-PAGE and fractions pure to homogeneity were pooled and analyzed by analytic gel-filtration using a Superdex 75 10/300 column (GE Healthcare) equilibrated in PBS, pH 7.4.

### 2.7 Kinetics of sdAb extraction from classical inclusion bodies

In order to study the kinetics of sdAb urea-mediated extraction from inclusion bodies, samples withdrawn at 0, 1, 2, 3, 4, 20, 22 and 24 h were analyzed by SDS-PAGE on a 15% acrylamide/bisacrylamide gel. Before loading, 12.5% (v/v) ice-cold trichloroacetic acid was added to the samples. After 15 min incubation at 4 °C, samples were centrifuged and the precipitated proteins were resuspended in 100 mM Tris–HCl pH 8.8.

### 2.8 Western-blot analysis

After the electrophoretic run, the proteins were transferred from within the gel to a 0.22 µm PVDF membrane (Merck-Millipore) using a semi-dry blotter system (SIGMA). After blocking with PBS-TB (1X Phosphate Buffered Saline pH 7.4, 0.1% v/v Tween 20, 1% w/v bovine serum albumin) the membrane was incubated with mouse anti-polyHistidine-peroxidase mAb (SIGMA, 1:7000 in PBS-TB) and washed twice with PBS-T. Signal was developed using an ECL system and detected by radiographic films. Relative bands quantification was performed using ImageJ software.
